# Emerging Roles of Ceramide in Cardiovascular Diseases

**DOI:** 10.14336/AD.2021.0710

**Published:** 2022-02-01

**Authors:** Hongyang Shu, Yizhong Peng, Weijian Hang, Na Li, Ning Zhou, Dao Wen Wang

**Affiliations:** ^1^Division of Cardiology, Department of Internal Medicine, Tongji Hospital, Tongji Medical College, Huazhong University of Science and Technology, Wuhan 430000, China.; ^2^Hubei Key Laboratory of Genetics and Molecular Mechanism of Cardiologic Disorders, Huazhong University of Science and Technology, Wuhan 430000, China.; ^3^Department of Orthopedics, Union Hospital, Tongji Medical College, Huazhong University of Science and Technology, Wuhan 430000, China

**Keywords:** ceramide, sphingolipid metabolism, cardiovascular disease, diagnostic biomarker, therapeutic target

## Abstract

Ceramide is a core molecule of sphingolipid metabolism that causes selective insulin resistance and dyslipidemia. Research on its involvement in cardiovascular diseases has grown rapidly. In resting cells, ceramide levels are extremely low, while they rapidly accumulate upon encountering external stimuli. Recently, the regulation of ceramide levels under pathological conditions, including myocardial infarction, hypertension, and atherosclerosis, has drawn great attention. Increased ceramide levels are strongly associated with adverse cardiovascular risks and events while inhibiting the synthesis of ceramide or accelerating its degradation improves a variety of cardiovascular diseases. In this article, we summarize the role of ceramide in cardiovascular disease, investigate the possible application of ceramide as a new diagnostic biomarker and a therapeutic target for cardiovascular disorders, and highlight the remaining problems.

## Introduction

Cardiovascular diseases (CVDs) impose a heavy economic and health burden worldwide [1-3]. According to a World Health Organization (WHO) report, CVDs are the most important lethal factors worldwide, accounting for 17.9 million deaths and representing 31% of total deaths globally in 2019. The number of individuals with CVDs is staggering, including those with heart failure, myocardial infarction, atherosclerosis, diabetic cardiomyopathy, lipotoxic cardiomyopathy, and hypertension [4-7]. Following the discovery of ceramide as the central signaling metabolite among sphingolipids, studies concerning its involvement in cardiac health and pathophysiology have increased exponentially.

Sphingosine was discovered and named by John L W Thudichum in the 19th century [8]. To date, over 4,000 different chemical entities in the sphingolipid family have been identified (www.lipidmaps.org/). Sphingolipids (ceramide, sphingomyelin, sphingosine, sphingosine-1-phosphate (S1P), and gangliosides) usually account for 2%-15% of total lipids, much lower than the abundance of glycerolipids [9]. Although they are relatively small, sphingolipids have many critical biological activities, such as changing the physical and chemical properties of the cell membrane’s double bilayer and modulating the activity of receptors [10, 11]. Among the various sphingolipids, ceramide is the central molecule. It comprises a fatty chain connected by sphingosine bases and amides, with lengths ranging from C14:0 to C26:0 [12]. The central role of ceramide in the sphingolipid pathway is mainly attributed to its involvement in synthesizing complex structural sphingolipids (sphingomyelin and ganglioside) and as the precursor of the survival molecule and immunomodulator, S1P [13].

In 1993, Obeid et al. demonstrated that tumor necrosis factor activates sphingomyelinase, thereby acutely releasing ceramide. This finding inspired researchers to investigate the role of ceramide as an intracellular messenger. Later, the ability of ceramide to induce apoptosis was confirmed [14, 15]. In 1998, Unger et al. showed that excessive ceramide results in the apoptosis of pancreatic β cells in obese Zucker diabetic fatty (ZDF) rats, indicating that ceramide is critically involved in metabolic abnormalities [16]. In addition, Summers et al. reported an intimate relationship between ceramide and insulin resistance: ceramide directly inhibits Akt activation and hormone-stimulated translocation of the insulin-responsive glucose transporter, GLUT4 [16]. The above two pioneering findings prompted researchers to focus on the role of ceramide in metabolic diseases.

It was not until 2005 that a link between ceramide and CVD was ascertained by the report that the inhibition of *de novo* ceramide biosynthesis in ApoE-KO mice prevented the development of atherosclerotic lesions that underlie various CVDs [17]. Since then, the application of pharmacological agents or transgenic mice to explore the role of ceramide in CVD has become a research hotspot. Inhibiting the synthesis of ceramide has been proven to prevent the development or reverse the progression of insulin resistance, atherosclerosis, hypertension, and cardiomyopathy [18-22]. Serum and tissue ceramides have been confirmed to be closely related to insulin resistance, diabetes, CVD incidence, secondary CVD events, secondary CVD mortality, and coronary artery disease, and the development of mass spectrometry and lipidomics technology has allowed researchers to comprehensively evaluate the accumulation of sphingolipids in serum and tissues [23, 24]. Therefore, ceramide may serve as an another new biomarker for predicting cardiovascular events [25, 26].

**Figure 1.**
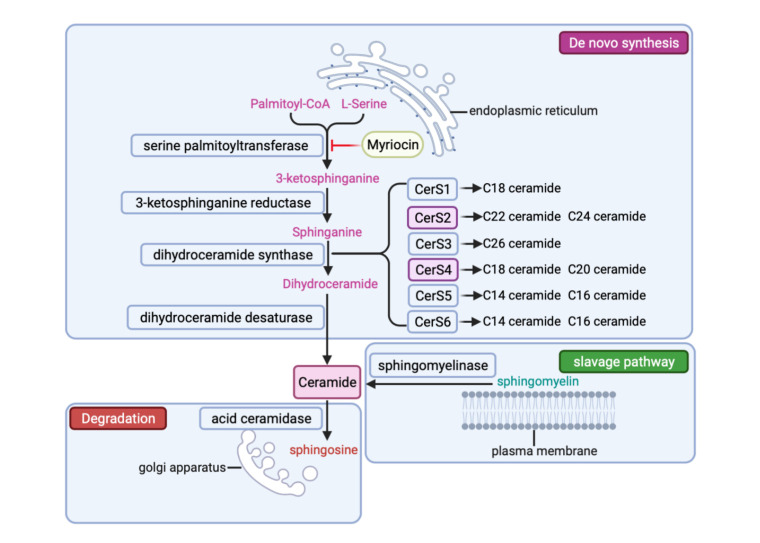
The metabolic pathway of ceramide. De novo synthesis (purple) takes place in the endoplasmic reticulum, L-serine and Palmitoyl-CoA are catalyzed by serine palmitoyltransferase (which has a specific inhibitor-Myriocin) to produce 3-Ketosphinganine, which is then converted to sphinganine by 3-Ketosphinganine reductase. Six kinds of CerSs (CerS4 and CerS2 dominates in cardiomyocyte) then further synthesize the sphinganine into dihydroceramide, which are then reduced by dihydroceramide desaturase to produce ceramides of different acyl chain lengths. Ceramide can also be generated through the slave pathway (green) on the cell membrane or be transported to the Golgi apparatus and degraded by acid ceramide into sphingosine.

In this review, we summarize the biochemical process of ceramide and its role in CVD to investigate the possible uses of ceramide as a new diagnostic biomarker and therapeutic target for cardiovascular disorders. Finally, we highlight the remaining problems and perspectives for future research.

## Ceramide metabolic pathway

### De novo synthesis of ceramide

The first step in the *de novo* synthesis of ceramide is the condensation of L-serine and palmitoyl-CoA to produce 3-ketodihydrosphingosine (KDS), which is catalyzed by serine palmitoyltransferase (SPT) [27] (Fig. 1). SPT is an enzyme located in the endoplasmic reticulum (ER). It is composed of a catalytic LCB1/LCB2 heterodimer and a small activated subunit (Tsc3 in yeast; ssSPTs in mammals), which is negatively regulated by the evolutionarily conserved family of Orosomucoid-like proteins (ORM). ORM combines with the Lcb1 transmembrane domain 1 of SPT to form a stable complex that regulates the activity and localization of SPT. This complex is also directly regulated by ceramides [28, 29]. Myriocin is a specific inhibitor of SPT [30-32]. Myriocin first combines the active site of pyridoxal 5-phosphate to form a complex that degrades into C18-aldehyde through a 'retro -aldol-like' cleavage mechanism. C18-aldehyde then acts as a suicide inhibitor of SPT by covalent modification of the essential catalytic lysine residue [33]. Intestinal flora encoding SPT, such as *Bacteroides* spp., produce ceramide, which enters the host's blood circulation through the hepatic portal venous system, and inhibits the *de novo* synthesis of ceramide [34].

Next, 3-ketosphinganine reductase converts KDS to sphinganine, which is then catalyzed by the CerS family proteins to generate dihydroceramide with variable acyl chain lengths (Fig. 1). In mammals, six CerSs, mainly localized in the endoplasmic reticulum, have been identified [35, 36]. In the heart, CerS4 had the highest expression level, followed by CerS2 [37]. CerS proteins are known to prefer specific fatty acyl substrates, thereby regulating the synthesis of dihydroceramides containing specific acyl chain lengths [36, 38]. CerS4 has unique selectivity for C18:0-CoA and C20:0-CoA as fatty acyl substrates, thereby generating C18:0 and C20:0 dihydroceramide, while CerS2 prefers C22:0, C24: 0, and C26:0, producing long-chain dihydroceramide [39]. Knockout of CerS1 in muscle significantly reduces C18:0 ceramide levels and improves whole-body glucose homeostasis. However, the specific knockout of CerS5 and CerS6 that produce C16:0 ceramide does not protect mice from obesity-induced insulin resistance, indicating different roles of different CerSs in specific tissues [40]. A recent study demonstrated that single nucleotide polymorphism (SNP) rs267738, causing partial loss of CerS2 function, enhances diet-induced glucose intolerance in mice but does not significantly affect human serum sphingolipids or influence the ceramide-based CERT1 CVD risk score [41]. Nevertheless, the function of CerSs in heart tissue remains unclear.

The last step of *de novo* synthesis of ceramide is introducing 4,5-trans double bonds in dihydroceramide by dihydroceramide desaturase to convert it into ceramide [42] (Fig. 1). Targeting the ceramide double bond improves insulin resistance and steatosis. Therefore, dihydroceramide desaturase has also become a potential therapeutic target against cardiometabolic diseases [18, 43]. Two dihydroceramide desaturase genes have been identified, *DES1* and *DES2*, which encode Des1 and Des2 proteins, respectively. Des1 is widespread and has high dihydroceramide C4-desaturase activity, while Des2 is mainly expressed in the small intestine, skin, and kidney [44]. Des2 plays an important role in the synthesis of phytoceramides and glycosphingolipids containing 4-hydroxysphingosine [45].

### Degradation of ceramide

The degradation of ceramide into sphingosine and free fatty acids is mainly mediated by ceramidase in the Golgi apparatus (Fig. 1). Five different ceramidases were identified and classified according to the optimal pH for their catalytic activity. They are acid ceramidase (pH= 4.5), neutral ceramidase (pH= 7.5), alkaline ceramidase 1 (pH=8.5), alkaline ceramidase 2 (pH=9.0), and alkaline ceramidase 3 (pH=9.5) [46]. In addition to these five classical ceramidases, adiponectin receptors have also been reported to have inherent basic ceramidase activity, enhanced by adiponectin [47-49]. Acid-ceramidase is dominant in the heart. Overexpression of acid ceramidase effectively reduces cardiomyocyte mortality and provides cardioprotection [50].

### Ceramide regeneration

The decomposition of sphingomyelin can also generate ceramide via acidic or neutral sphingomyelinase in the plasma membrane, referred to as the salvage pathway [51, 52] (Fig. 1). Magnesium deficiency promotes the occurrence of CVDs; the use of phorbol-12-Myristate-13-Acetate stimulation released ceramide but not sphingosine, suggesting a role for the "sphingolipid salvage pathway" in magnesium vascular muscle [53]. Intestine HIF-2a inhibition markedly reduced intestinal and serum ceramide levels through the salvage pathway by positively regulating the expression of neuraminidase 3 [54].

## Ceramide and CVDs

### Ceramide and myocardial infarction

**Figure 2.**
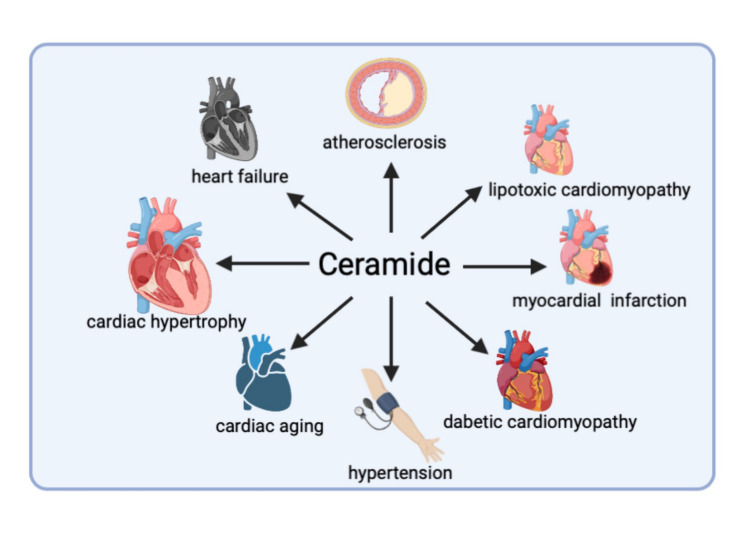
Ceramide accumulation in cardiovascular system has been implicated in the impairment of many pathological processes that underlie cardiovascular diseases. The figure illustrates key diseases associated with ceramide accumulation.

In patients with ST-segment elevation myocardial infarction (STEMI), ceramide levels in extracellular vesicles are significantly increased before reperfusion [55]. Ceramide levels were also significantly increased in the left ventricle of patients with chronic ischemia (Fig. 2). The main types of ceramides with increased abundance are C16, C20, C20:1, and C24 [50]. They are positively correlated with plaque rupture and the severity of coronary artery stenosis in patients with STEMI [56, 57]. Therefore, ceramide is used as a biomarker and an independent diagnostic indicator in the prognosis of acute myocardial infarction and has been proven to have great potential in combination with high-sensitivity troponin T in identifying patients with acute coronary syndrome [58]. The recently developed cardiovascular event risk test 2 (CERT2) based on ceramide and phospholipids effectively detects the risk of death in patients with stable coronary heart disease. Hazard ratios per standard deviation for the CERT2 risk score were 1.23 (95% CI, 1.14-1.33) for myocardial infarction when adjusted for traditional cardiovascular risk factors [59].

Activation of the sphingomyelin hydrolysis pathway and *de novo* synthesis pathway contributes to the increase in ceramide. Several genes related to *de novo* synthesis of ceramide are upregulated in myocardial infarction, including serine palmitoyltransferase, acid sphingo-myelinase, and neutral sphingomyelinase [60, 61]. The activity of the secretory acid sphingomyelinase is also significantly enhanced in patients with unstable angina and acute myocardial infarction [61]. Short-hairpin RNA (shRNA) targeting neutral sphingomyelinase isoform 3 prevents ceramide elevation caused by myocardial infarction in rats [62].

The increase in ceramide levels in cardiac tissue is related to higher cardiomyocyte mortality and deterioration of cardiac function [50]. Inhibiting the sphingomyelin hydrolysis pathway or *de novo* synthesis pathway to reduce ceramide alleviates ventricular remodeling and improves cardiac function. Delivery of modified acid neuraminidase (AC modRNA) enhances AC activity and reduces ceramide levels, which effectively lowers cardiomyocyte mortality and reduces pro-inflammatory and harmful neutrophils in the left ventricle, thereby achieving cardioprotection after myocardial infarction [50]. Blocking SPT with the specific inhibitor, myriocin, reduces C16, C24:1, and C24 levels of ceramide and improves ventricular remodeling and fibrosis after myocardial infarction [60]. Diaphragm weakness and decreased ejection fraction in rats with heart failure caused by myocardial infarction are ameliorated in the context of reduced levels of neutral sphingomyelinase or lowered neutral sphingomyelinase activity by shRNA or a pharmacological inhibitor (GW4869) [62].

Ceramide also aggravates damage to the ischemic heart by promoting inflammation. A cross-sectional analysis of 33 patients with coronary heart disease showed that the concentration of serum ceramide is closely related to serum IL-6 levels [63]. Moreover, there is a strong positive correlation between lipid metabolites related to the sphingolipid pathway, sphingomyelin and ceramide, and acute inflammation markers (high-sensitivity C-reactive protein) [64]. This evidence indicates that ceramides may facilitate inflammation in coronary heart disease.

### Ceramide and cardiac hypertrophy

Cardiac hypertrophy is characterized by cardiomyocyte hypertrophy and an independent risk factor for heart failure and other CVDs. In age-related myocardial hypertrophy and myocardial hypertrophy caused by pressure overload, increased ceramide levels can be observed (Fig. 2), which may be due to the inhibition of peroxisome proliferator-activated receptor alpha (PPARα) [65]. Depending on the activation of ceramide synthase 5, a western diet rich in saturated fatty acids induces myocardial hypertrophy and left ventricular diastolic dysfunction in mice [66]. Western diet feeding leads to inappropriate accumulation and redistribution of ceramide substances in the heart, further aggravating heart remodeling in mice with cardiac hypertrophy caused by pressure overload [67].

The protective effects of various molecules and drugs on pathological myocardial hypertrophy depend on the modulation of ceramide, the overexpression of fat triglyceride lipase (ATGL), the rate-limiting enzyme of triglyceride (TG) hydrolysis, increases PPARα activity, which results in the enhancement of FA oxidation, reduction of ceramide, and prevention of pressure overload-induced cardiac hypertrophy [68]. Acyl-coenzyme A synthetase 1 (ACSL1) plays a vital role in the absorption of long-chain fatty acids (LCFA) and the activation of coenzyme A (CoA). The cardiac function of TAC mice with *ACSL1* knockout was much better than that of non-transgenic mice [69]. Its protective effect relies on the inhibition of *de novo* synthesis of cardiotoxic C16 and C24- and C24:1 ceramide. The deficiency of carnitine palmitoyltransferase-1b (CPT1b), a mitochondrial β-oxidation rate-limiting enzyme that controls the uptake of long-chain acyl-CoA in the mitochondria, results in myocardial lipid accumulation, increased ceramide levels, and worsened pathological myocardial hypertrophy in the cardiac pressure overload model [70]. Non-targeted metabolomics and lipidomics analysis also suggest that Huanglian Jiedu decoction combats pathological myocardial hypertrophy and remodeling induced by TAC via the reduction of lipids, such as ceramide [71].

### Ceramide and hypertension

Hypertension, defined as an increase in arterial blood pressure caused by abnormal contraction/dilation, is also the basis of myocardial infarction, hypertrophic cardiomyopathy, and heart failure [72, 73]. Ceramide was significantly increased in the arterial tissues of spontaneously hypertensive rats (SHRs) (513±19 pmol WKY vs. 645±25 pmol SHR, n?=?6-12, p<0.05) and in the plasma of patients with essential hypertension (185±8 pmol vs. 252±23 pmol; n?=?18 normotensive vs. n?=?19 hypertensive patients, p<0.05); the latter is positively correlated with the level of hypertension [74]. In an Uppsala elderly cohort study, ceramide was associated with longitudinal changes in diastolic blood pressure [75]. Lowering blood pressure is often accompanied by a reduction in ceramide levels. The level of ceramide in the blood vessels declines when blood pressure is lowered by the angiotensin II receptor antagonist, losartan, or the vasodilator, hydralazine [76]. Serum metabolomics based on liquid chromatography-tandem mass spectrometry (LC-MS) showed that blood pressure reduction effect of Uncaria relies on a decrease in ceramide levels [77]. In addition, the negative correlation between the intake of whole grains, edible fish oil, and blood pressure may be attributed to ceramide [78].

Endothelial dysfunction and vascular remodeling are hallmarks of hypertension [79, 80]. Endothelial nitric oxide synthase (eNOS) has a strong vasodilation effect [81]. In endothelial cells, ceramide disrupts the Akt-Hsp90-eNOS complex by activating PP2A, weakening endothelial-dependent vasodilation, which underlines endothelial dysfunction [82]. In addition, the extensive intermolecular hydrogen bonding between ceramide increases the stiffness of the membrane and reduces its fluidity [82]. Recent mechanistic studies have analyzed the role of specific ceramides in blood vessels. C16:0-Cer plays a major role in maintaining vasodilation induced by tyrosine kinases and G protein-coupled receptors (GPCRs). C24:0- and C24:1-Cer controls blood flow-induced vasodilation [83].

### Ceramide and heart failure

Apoptosis, inflammation, and stress responses in which ceramides are involved are all pathogenic factors of heart failure. Increased ceramide levels in the myocardium and serum of patients with advanced heart failure are linked to an increase in the rate-limiting enzyme (SPT) in the *de novo* ceramide synthesis pathway (Fig. 2). Blocking SPT with the specific inhibitor, myriocin, reduces ceramide levels in the myocardium and relieves heart remodeling [60]. Consistently, knocking out the cardiomyocyte Krüppel-like factor (KLF)-5 inhibits the expression of two critical factors (SPTLC1 and SPTLC2) that regulate the *de novo* synthesis of ceramide, thus preventing the accumulation of ceramide. Consequently, cardiac remodeling is inhibited, and the ejection fraction increases [31].

In a follow-up study with an average period of 4.4 years in 423 patients with heart failure, Yu et al. found that plasma ceramide levels increase and are related to cardiac inflammation in heart failure. Furthermore, ceramide has been proven to be an independent risk factor for death in patients with chronic heart failure and reduced left ventricular systolic function [31]. In particular, higher levels of ceramide d18:1/24:0 were associated with increased mortality in patients with chronic heart failure [84]. In routine risk assessment for cardiovascular diseases, ceramide may have additional predictive value. A study involving 2,652 Framingham offspring participants regarding ceramide and heart function revealed that higher levels of C16:0/C24:0 are associated with lower left ventricular ejection fraction, poor overall circumferential strain, higher left ventricular end-systolic volume, and lower left ventricular ejection fraction after adjusting for standard risk factors, including lipid markers, etc. [85]. Therefore, certain types of ceramides are robust risk factors for unfavorable cardiac pathology independent of lipid markers.

However, there is a discrepancy in the contributions of different types of ceramides to the risk of heart failure. In a study with a median follow-up of 9.4 years and 4,249 study participants, higher plasma levels of Cer-16 were associated with an increased risk of heart failure; in contrast, Cer-22 was associated with reduced risk [86]. In addition, in the TOPCAT trial, Cer16:0, 18:0, and 18:1 were associated with an increased risk of death or admission in heart failure patients, among which 16:0 showed the most significant correlation. However, Cer-24:0, which is considered to be associated with all-cause mortality, exhibited no relationship with the risk of death or hospitalization [87]. The above facts suggest that we need to identify further and distinguish the functions of different types of ceramides and unveil the underlying mechanism.

### Ceramide and atherosclerosis

Ceramide is related to the development of atherosclerosis, as evidenced by the significantly higher concentration of ceramide in aortic plaques (Fig. 2). In pigs with familial hypercholesterolemia, the size of LDL aggregates depends on the ceramide concentration [88, 89]. Cholesterol, triglycerides, and apolipoprotein B-100 proteins are positively correlated with ceramide concentration [90]. An omics analysis of plasma lipids in a multicenter HIV cohort showed that atherosclerotic plaques in persons infected with HIV had higher levels of ceramide (16:0) than those in persons without HIV infection [91]. Moreover, multi-omics analysis of the intestinal microbiota of 161 patients with coronary artery disease revealed that intestinal microbes might affect the occurrence of atherosclerosis by regulating the metabolism of ceramide [92]. Ceramide has been identified as a subclinical marker of atherosclerosis and is considered useful for assessing the risk of atherosclerotic CVD that exceeds the standard risk factors [93]. Hazard ratios for Cer16:0, Cer18:0, and Cer24:1 and atherosclerotic cardiovascular outcomes in different clinical trials range from 1.1(95%CI, 1.02, 1.21) to 4.49 (95%CI, 2.24-8.99). Risk scores CERT1 calculated from Cer(d18:1/16:0), Cer(d18:1/18:0), and Cer(d18:1/24:1) concentrations and their ratios to Cer(d18:1/24:0), and CERT2, which adds phosphatidylcholines to CERT1, have been developed and used for the primary and secondary prevention of atherosclerotic CVD [94].

Oxidized LDL (oxLDL) is the main lipid in atherosclerotic lesions. Elevated plasma oxLDL levels are a critical risk factor for atherosclerosis [95]. Endogenous ceramide facilitates oxLDL transport across endothelial cells and promotes its retention in the endothelium of the blood vessel wall. Reducing the production of endogenous ceramide by the acid sphingomyelinase inhibitor desipramine (DES) and the *de novo* ceramide synthesis inhibitor (myriocin) significantly inhibits the transcellular effect of oxLDL [96, 97]. Ceramide also promotes the formation of foam cells by impairing the digestion of aggregated LDL (agLDL) via macrophages [98].

Leptin resistance in endothelial cells leads to vascular endothelial dysfunction, which marks the onset of atherosclerosis. The downregulation of acid sphingomyelinase improves endothelial leptin resistance and atherosclerosis [99]. Ceramide also plays an indispensable role in the transition of blood flow-induced dilation (FID) mediators from nitric oxide (NO) to mitochondrial-derived H_2_O_2_ [100], inhibiting the production of ceramide via myriocin restores the FID mediator to nitric oxide, elevates NO bioavailability, and reverses atherosclerosis [101].

Atherosclerosis is a chronic inflammation of the blood vessel wall. Ceramide has been proven to promote the production of IL-6 and C-reactive protein (CRP), which have direct pro-inflammatory effects in the atherosclerotic process [102]. TNF-α assembles in endothelial cells and leads to endothelial dysfunction [103]. TNF-α is an important factor for ceramide accumulation in endothelial cells [104]. Accompanied by reactive oxygen species (ROS), TNF-α stimulates ceramide formation by activating neutral and acidic sphingomyelinase. In addition, C2-ceramide, a highly cell-permeable ceramide analog, has been shown to stimulate human umbilical vein endothelial cells to express TNF-α [105, 106]. Ceramide and TNF-α may enhance the effects of inflammation on atherosclerosis through this vicious cycle.

### Ceramide and diabetic cardiomyopathy

Diabetic cardiomyopathy (DCM) is a unique form of heart disease caused by diabetes and is different from traditional factors. It is characterized by myocardial metabolic remodeling [107], in which lipid overload appears in the early onset of diabetic cardiomyopathy, which is also the primary cause of myocardial hypertrophy in diabetic cardiomyopathy [108, 109]. Accumulation of the toxic metabolite ceramide has been observed in diabetic cardiomyopathy mice [110] (Fig. 2). Ceramide neutralizes the effect of insulin by inhibiting Akt, reducing insulin-stimulated GLUT4-mediated glucose uptake, and further aggravating lipid accumulation [111]. In the myocardial mitochondria of type I diabetic mice, lactose ceramide effectively inhibited mitochondrial respiration and reduced calcium retention capacity, leading to mitochondrial dysfunction [112]. In addition, ceramide synthase 5 has also been confirmed to mediate lipid-induced autophagy in cardiomyocytes [66]. A recent pilot study showed that a diet rich in medium-chain fatty acids improves the contractile function of patients with type 2 diabetes and changes their liposome composition, including lowering the level of ceramide, which underscores ceramide as a therapeutic target[113]. The above evidence emphasizes the damaging effect of ceramide on the heart under lipid overload, but more underlying mechanisms and therapeutic strategies remain to be elucidated.

## Ceramide and lipotoxic cardiomyopathy

Lipotoxic cardiomyopathy is the result of an abnormal accumulation of myocardial lipids, including ceramides [114]. Increased levels of myocardial ceramide are associated with diastolic dysfunction in Akita Ins2 (WT/C96Y) and Zucker diabetic fat (ZDF) rats [115, 116]. Moreover, leptin-induced suppression of cardiomyocyte contraction is amplified by ceramide [117]. Excess fatty acids are synthesized into triglycerides and ceramides when fatty acid intake and oxidation are out of balance [12, 118]. Reducing the uptake of fatty acids in the heart by the PPARg agonist troglitazone and insulin and promoting the conversion of fatty acids into non-toxic triglycerides by overexpression of diacylglycerol acyltransferase 1 lowers the level of ceramide and improves heart function [119]. Reducing ceramide by inhibiting SPT in the isolated heart perfused with LpL (GPI) also improves its contractile function and increases its survival rate, indicating that ceramide plays a critical role in lipotoxicity cardiomyopathy [120]. Ceramide-related hyperlipidemia is partly mediated by caspase activation, sarcomere maintenance, and lipogenesis because cardiac cells specifically knock out the caspase activator Annexin-X and myosin chaperone Unc-45, lipogenic enzyme FASN1, and other ceramide-interacting proteins prevent lipotoxic cardiomyopathy [22]. In addition, excess ceramide promotes apoptosis and leads to the loss of cardiomyocytes [121, 122]. However, increased cardiomyocyte apoptosis was not observed in the myocardium of ob/ob mice, and rats fed a high-fat diet, even when the ceramide level increased. This may be related to the insufficient concentration of ceramide and the observation time [123]. Recent studies have reported that the effect of lipotoxic ceramide on mitochondrial function is chain length-dependent. Inhibiting the increase in VLC ceramides produced by CerS2 can improve mitochondrial autophagy and oxidative stress. However, ceramide produced by CerS5 has no effect, suggesting that distinguishing the type and source of ceramide may facilitate the precise treatment of lipotoxic cardio-myopathy [124].

## Ceramide and cardiac aging

Cardiac aging is an age-related pathological change that causes a series of functional and structural alterations, thereby increasing the risk of cardiovascular disease in the elderly [125, 126]. Age-related ceramide accumulation in the aging heart is closely associated with cardiolipin decline and cardiac mitochondrial dysfunction promotion. Compared with accelerated aging resistance mice (SAM-R1), accelerated aging mice (SAM) developed cardiac hypertrophy, which may be a result of increased ceramide in the heart[65]. Three subtypes of ceramide (C(16)-, C(18)-, and C(24:1)-ceramide) increase in the mitochondria of the aging rat heart. This increase may be due to the increased hydrolysis of sphingomyelin caused by neutral sphingomyelinase, whose activity increases with age[127]. Reducing mitochondrial ceramide levels by administration of (R)-α-lipoic acid (LA) can reverse the age-related decline in glutathione levels and increase the activity of complex IV, thus improving mitochondrial function[127].

## Ceramide and ischemia-reperfusion

Ischemia-reperfusion is defined as a sudden interruption of blood flow in the heart vessels. After blood flow is restored, heart tissue damage is aggravated. In 1997, Bielawska et al. first observed the accumulation of ceramide in cardiac ischemia-reperfusion injury (Fig. 2) [128]. Subsequently, Beresewicz et al. confirmed that seven of the 14 ceramides identified in the heart (ceramides containing palmitic acid, stearic acid, oleic acid, linoleic acid, and arachidonic acid) increased by different magnitudes, independent of their basal tissue concentrations [129]. The above seven ceramides also account for the hemodynamic improvement of ischemic preconditioning. Moreover, ceramides (Cer(d18:0/16:0) and Cer(t18:0/12:0)) have high sensitivity and specificity in the prognostic prediction of major adverse cardiovascular events, according to the plasma metabolic profile of young patients with STEMI [130].

One of the earliest responses of cardiomyocytes to hypoxia and reoxygenation is the activation of neutral sphingomyelinases [131]. Treatment with the sphingomyelinase inhibitor, D609, inhibits neutral sphingomyelinase activity, reduces ceramide accumulation, and effectively alleviates ischemia-reperfusion injury [132]. After transient coronary artery occlusion in mice, ceramide synthesis increases during reperfusion in the ischemic area (at-risk region) surrounding the necrosis. This is related to the increased expression of the rate-limiting enzyme (SPT) in the *de novo* synthesis pathway [30]. In ischemia-reperfusion, excessive ROS is generated, and a large number of inflammatory cytokines, including TNF, are recruited in the process of reperfusion involving the ischemic area. Several studies have shown that ceramide aggravates ischemia-reperfusion injury by promoting cell death and tissue damage induced by ROS and TNF-?? [133]. Therefore, the inhibition of *de novo* synthesis and sphingomyelin hydrolysis pathways to reduce ceramide synthesis has become a treatment modality for ischemia and reperfusion. For example, the SPT inhibitor, myriocin, significantly protects the ischemic area surrounding necrosis from damage [134]. In the ischemic area surrounding necrosis, myriocin downregulates ceramide, reduces inflammation and other mediators of ROS, and activates Nrf2-HO1 protective responses [30].

In addition, the protective effect of cardiac ischemic preconditioning or desipramine (a sphingomyelinase inhibitor) on ischemia-reperfusion is partly attributed to the inhibition of ceramide generation [134, 135]. Control ischemia-reperfusion hearts exhibited a peak of ceramide production at 5 min of prolonged ischemia, with a mean value averaging 64 ± 5 ng/mg tissue (P < 0.05, vs. 48 ± 4 ng/mg at baseline). In contrast, ischemic preconditioning prevented ceramide increase during prolonged ischemia with less apoptosis, as well as a limited infarct size.

## Concluding remarks and future perspectives

Globally, aging has accelerated the prevalence of CVD [3]. Thus, there is an urgent need to identify more effective molecular targets. It took more than 100 years to identify sphingomyelin in 1884 to confirm the close connection between ceramide and atherosclerotic plaque formation in 2005. With the rapid development of lipidomics and transgenic mouse technology, ceramide has been identified as a potential diagnostic biomarker of major adverse cardiac events and a molecular target in the treatment of CVDs in the last 20 years. Decreasing ceramide levels through pharmacology and gene modification of the enzymes involved in the synthesis and degradation of ceramide are becoming new strategies. However, the following problems persist:

First, although lipidomics and mass spectrometry have determined the relationship between plasma ceramide and cardiometabolic dysfunction, there is still a lack of multicenter epidemiological investigations to further verify the role of different types of ceramides in predicting major adverse cardiac events.

Second, lowering ceramide levels helps to impede the onset and progression of metabolic CVDs. However, the heart-specific deletion of the two alleles of *Sptlc2* results in abnormal heart development. The effects of ceramide on cardiac development are different from those on metabolic heart disease, conferring various roles of ceramide in CVDs [136].

Third, the specific mode of ceramides is not clear. For example, although several mechanisms, including inflammation and endothelial dysfunction, have been identified to explain the action of ceramide in atherosclerosis, they cannot explain all of the benefits of ceramide depletion. ROS accumulation, mitochondrial autophagy, ER stress, etc., may also contribute to the protective effects of ceramide reduction. Therefore, it is necessary to explore the underlying mechanisms of ceramides.

Finally, an interesting missense mutation (rs267738) in the ceramide-modifying gene, *CERS2*, was confirmed to be closely associated with HbA1c [137]. The rs267738 SNP causes partial loss of CERS2 function, thereby worsening the metabolism of knock-in mice [41]. Revealing links between genetic polymorphisms of related enzymes in the ceramide pathway and CVDs would be beneficial for assessing the risk of CVDs in subpopulations to implement early warning and preventive treatment for high-risk groups. However, studies focusing on genetic associations are still in their infancy. Moreover, rs267738 is significantly related to metabolism in rodents, but it is not sufficient to affect the serum sphingolipid profile of subjects with coronary artery disease in Utah [41]. This finding emphasizes the differences between rodents and humans, suggesting that researchers should be concerned about the transformation of rodent research into the human body.

In general, although there remain problems to be further studied, a large amount of evidence has confirmed the crucial role of ceramide in the development of CVDs. Regulating ceramide metabolism helps to relieve a variety of CVDs, including atherosclerosis, hypertension, myocardial infarction, ischemia-reperfusion, and heart failure. Taking ceramide as a core regulatory target may have a broad potential to combat CVDs.
